# Prioritization of operational research questions on COVID-19 vaccination in the African Region

**DOI:** 10.11604/pamj.supp.2022.41.2.33821

**Published:** 2022-03-29

**Authors:** Balcha Masresha, Ado Bwaka, Richard Mihigo

**Affiliations:** 1 World Health Organisation, African Regional Office, Brazzaville, Congo,; 2 World Health Organisation, African Regional Office, Inter-country Support team for Western Africa, Ouagadougou, Burkina Faso

**Keywords:** COVID Vaccination, operational research, research priorities, African Region

## Abstract

**Introduction:**

a year after the start of COVID-19 vaccination, coverage remains very low in the African Region. Different challenges and operational barriers have been documented, but countries will need to supplement the available information with operational research in order to adequately respond to practical questions regarding how best to scale up COVID-19 vaccination. We conducted a survey among immunisation program staff working in the African Region, in order to identify the high priority operational research questions relevant to COVID-19 vaccination.

**Methods:**

proposed operational research questions categorized into six topic areas were sent to resource persons, asking them to rate according to the relevance, urgency, feasibility, and potential impact of the research questions on the progress of COVID vaccination.

**Results:**

a total of 25 research questions have been given an average weighted rating of 75% or more by the respondents. Nine of these top priority research questions were in the area of demand generation, risk communication and community engagement while 8 questions covered the area of service delivery.

**Conclusion:**

countries should plan for and coordinate stakeholders to ensure that relevant operational research is done to respond to the top priority research questions, with a view to influence policies and implementation of strategies.

## Introduction

The outbreak of SARS-CoV-2, that causes COVID-19 disease has been spreading globally since the viruses were first detected in December 2019. On 11 March 2020, the World Health Organisation (WHO) declared a global pandemic [1]. The implementation of case management and non-pharmaceutical public health interventions to contain spread of the virus have been ongoing since the start of the epidemic. Alongside these measures, the development of multiple COVID-19 vaccines was fast-tracked. As of 11^th^ February 2022, ten types of vaccines have received WHO Emergency Use Listing, while there are 142 candidates vaccines in clinical development, and 195 vaccines products in pre-clinical stages of development [2,3]. The first countries in the WHO African Region started administering COVID-19 vaccination to priority groups in February 2021, and 46 countries are administering the vaccines as of February 2022. However, only 6.8% of the population in the Region has received full COVID-19 vaccination so far, and only 5 countries attained 40% coverage by December 2021 [4].

Countries in the African Region have long-standing experience with mass vaccination activities for the control of different vaccine preventable diseases, including poliomyelitis, measles, yellow fever, meningococcal meningitis, neonatal tetanus, and Ebola. Most of the experiences and lessons from these vaccination campaigns are transferable for use during COVID-19 vaccination, since the basics of the vaccination interventions are similar [5]. However, in the case of the control of COVID-19, multiple new vaccines with different product characteristics were introduced over the past year, targeting primarily adult populations, thus requiring the adaptation of new operational approaches [6].

By December 2021, 26 countries in the African Region conducted Intra-Action Reviews (IARs) to assess the vaccination response to the pandemic, using a standard protocol [7]. According to these IARs and field observations, some of the critical operational challenges faced at country level include the multiplicity of new vaccines introduced within a very short period of time, the short shelf-life of some of the COVID-19 vaccines (specifically AstraZeneca, Pfizer and Moderna vaccines), the shortage of and erratic supply of vaccines, shortage of operational funding leading to limited preparations and limited scope of service delivery, as well as weak public demand for vaccines in the face of infodemics [8]. Various studies have also documented similar challenges [5,9]. While these are the common findings across many countries, the experiences and specific challenges faced differ among countries, and even within countries.

In order to address the specific operational barriers and successfully scale up the vaccination response, countries will have to better understand what works and what has not worked within their own contexts. In this regard, the conduct of relevant operational research is critical. In the first year of COVID-19 vaccine roll out, most of the published research output on COVID-19 vaccines in the African context has been on the public perceptions and hesitancy to COVID-19 vaccination. Hence, there is a need for researchers to look into other program domains like coordination, service delivery, logistics, vaccine safety and monitoring, etc in order for the vaccination response teams to generate innovative and tailored solutions to local challenges.

In 2021, Africa CDC and WHO AFRO conducted a research prioritization exercise and identified major programmatic questions that need to be addressed in the response to the pandemic [10]. However, this research prioritization exercise did not address the vaccination pillar of COVID-19 response. The vaccination area of work is quite broad, and any prioritization of research questions will have to help researchers and donors make the best possible selections in terms of using scarce resources, and eventually help guide policy decisions. There are multiple criteria that can be used in the prioritisation of research questions. The contexts in which the priority setting is done often define the criteria to be used. Rudan *et al*. have defined some of these criteria for priority setting in health research [11].

Our study aims to shed light on the high priority operational research questions relevant to COVID-19 vaccination, in order to guide national public health program managers, academic and research institutions. In addition, such a prioritization exercise will help guide research investment into responding to practical issues that can improve the quality and equity of interventions.

## Methods

Based on their experience with country support in the first 12 months of COVID-19 vaccine introduction, earlier work with immunisation programs in the African Region, and also through formal and informal discussions within the WHO African Regional immunisation program, the investigators identified 62 operational research questions by topic area. These research questions were categorized into six research topic areas: 1) planning and coordination, 2) service delivery, 3) demand generation, risk communication and community engagement, 4) vaccine safety and adverse events following immunisation (AEFI) surveillance, 5) monitoring and data analytics, 6) cold chain and vaccine handling.

The questionnaire with the proposed research questions were e-mailed to resource persons identified from the country, subregional and African Regional level, including WHO staff, consultants and national immunisation program managers. The participants were requested to rate each of the proposed research questions on a scale of 1-5, according to 4 criteria: relevance, urgency, feasibility, and potential impact. These criteria were selected in order to simplify the process, considering the emergency context and the operational nature of the research questions to be prioritised. A weighting score was assigned to these criteria, and a composite percentage score was generated for each question based on the rating provided by the respondents. In addition, respondents were requested to provide additional high priority research questions that were not listed specifically in the survey. The data analysis was done using MS-Excel. Frequency statistics were used to describe the findings.

## Results

The survey questionnaire was e-mailed to a total of 111 resource persons, of whom 54 (49%) have filled and submitted the survey on time. Twenty-nine of these respondents were national immunisation program managers and WHO immunisation staff based in 22 countries in the African Region of the WHO, while the remaining 25 respondents were WHO immunisation program staff and consultants from the subregional and the African Regional level.

The research questions that received the highest rating are shown in Table 1. A total of 25 research questions have been given an average weighted rating of 75% or more. The majority of these top priority research questions were in the area of demand generation, risk communication and community engagement (9 questions) and service delivery (8 questions). The top two planning and coordination questions with the highest ratings were: 1) what is the role and the impact of full-scale mass-vaccination campaigns in COVID-19 vaccination? 2) What is the operational cost of delivering COVID-19 vaccination services in various countries and in various contexts?

**Table 1:** priority research questions with > 75% weighted rating

**1. Planning and coordination**	
What is the role and the impact of mass-vaccination campaigns for COVID-19 vaccination?	81%
What is the operational cost of delivering COVID vaccination services in various countries and in various contexts?	77%
**2. Service delivery**	
What are the barriers to service delivery and high uptake of COVID-19 vaccines in countries at different levels of immunisation program maturity?	88%
What have countries done to maintain the balance between COVID-19 vaccination and the routine childhood / adolescent immunisation services?	85%
What are the optimal service delivery approaches to improve COVID-19 vaccine uptake among the general population?	83%
What are practical issues faced by health workers when handling multiple types of COVID-19 vaccines at the operational level?	82%
What types of routine services have been used to integrate COVID vaccination and how successful are these integration efforts?	80%
What are the optimal approaches to reaching hard-to-reach populations with COVID vaccination?	78%
What are the various regulations and mandates implemented by countries to increase COVID-19 vaccine uptake?	76%
What is the level of client acceptance of integrating COVID vaccination with different routine health care services?	76%
**3. Demand generation, Risk Communication and Community Engagement**	
What is the magnitude of hesitancy (reservation and / or refusals) to COVID vaccines among different population sub groups. What are the factors related to it, and how has it changed with time?	88%
What are the major behavioral drivers related to vaccine hesitancy among different population subgroups, and in different contexts, by gender, by age group, by socio-economic status?	84%
What is the public perception regarding the safety of COVID vaccines, and adverse events following COVID vaccination?	83%
What reminder systems are best suited to improve uptake of second dose COVID vaccines?	83%
What are the most trusted sources of information used by different population groups for information about COVID vaccines and vaccination?	82%
What approaches do different countries utilize for rumor and infodemic tracking, and which approaches are most efficient in terms of timely monitoring the trends?	82%
What are the reasons for defaulting after the first dose of COVID vaccination?	80%
What is the knowledge attitude and practice towards COVID vaccines and vaccination among the general population, and different population subgroups ( eg, health workers, teachers, college students, urban vs rural residents, different age groups, different socio-economic groups, etc)?	80%
Are mass messaging platforms (e.g. WhatsApp) and social media effective tools for COVID-19 vaccine demand generation?	78%
**4. Vaccine safety and AEFI surveillance**	
What are the most common types of AEFIs following COVID vaccines and their prevalence by type of vaccine?	76%
**5. Monitoring and data analytics**	
What systems are countries using to provide secure and authentic vaccination records?	78%
What is the role of digital tools (eg., mobile electronic tools) for data capture and real time monitoring during COVID-19 vaccination?	77%
**6. Cold chain and vaccine handling**	
What are the cold chain challenges related to COVID vaccines and vaccination in countries at different levels of immunisation program maturity?	77%
What is the additional cold chain space required for different types of COVID vaccines?	76%

In the area of service delivery, the following two research questions were rated highest by the respondents: 1) what are the barriers to service delivery and high uptake of COVID-19 vaccines in countries at different levels of immunisation program maturity? 2) What have countries done to maintain the balance between COVID-19 vaccination and the routine childhood / adolescent immunisation services? With regards to demand generation and communication, more research questions have been identified with rating of 75% and above, than in other topic areas. The top rated two questions were the following: 1) what is the magnitude of hesitancy to COVID-19 vaccines among the general public, and what are the factors related to it? 2) What are the major behavioral drivers related to vaccine hesitancy among different population subgroups, and in different contexts, by gender, by age group, by socio-economic status?

In the area of vaccine safety and AEFI surveillance, the respondents gave a rating of 75% or more to only one question: What are the most common types of AEFIs following COVID vaccines and their prevalence by type of vaccine? Monitoring and data analytics was another topic area, and here, the respondents gave a rating of 75% or more to these questions. 1) How do COVID vaccination coverage levels differ among persons of different socio-economic characteristics (age group, income groups, educational status, gender, religion, residence, etc)? 2) What is the role of digital tools (eg. mobile electronic tools) for data capture and real time monitoring?

Looking at cold chain and vaccine logistics, the following questions received ratings of 75% or more: 1) what are the cold chain challenges related to COVID-19 vaccines and vaccination in countries at different levels of immunisation program maturity? 2) What is the additional cold chain space required for different types of COVID-19 vaccines? The study respondents also proposed a few more research questions that were not in the initial list. These include:

**Planning and coordination:** 1) what is the basis for health budget execution (including the budget for COVID-19 vaccination) in relation to COVID-19 funding flows in different countries? 2) What are the urgent training needs for health workers involved in COVID-19 vaccination?

**Service delivery:** 1) what is the effectiveness of different service delivery approaches (routine service delivery, campaigns, mixed approaches) in terms of reaching the targets for COVID-19 vaccination? 2) How can active follow-up of clients for adverse events following vaccination improve vaccine uptake? 3) What is the impact of COVID-19 vaccine introduction on routine immunisation financing?

**Demand generation and communication:** 1) what are the factors related to specific COVID-19 vaccine product preferences? 2) How has vaccine hesitancy changed with time among different population groups? 3) How does the availability of multiple vaccine brands impact the vaccine acceptance and uptake in the communities?

**Logistics and vaccine handling:** what is the impact of using COVID vaccines without vaccine vial monitors (VVM) on the handling and potency of the vaccines at the point of use?

**Vaccine safety and AEFI surveillance:** what is the cost-effectiveness of the different approaches of AEFI surveillance in different contexts?

## Discussion

Despite the availability of multiple COVID-19 vaccine products within a relatively short period of time after the start of the pandemic, vaccination acceptance and coverage levels remain low in the African Region. National program managers and stakeholders will need to pinpoint the challenges and the root causes, and generate solutions best suited to their contexts in order to scale up vaccination [12]. Progress towards high COVID-19 vaccination coverage levels is dependent on agile approaches, learning lessons and making the necessary adjustments to implementation as needed [9,13,14]. The design and implementation of quick and practical operational research projects is critical to better understand the realities at the local level, and to refine strategies in a dynamic manner. Various authors have argued for the need for better coordination and accelerated implementation of research on COVID-19, especially in low resource settings [15]. A large number of funded research projects are underway in the broad area of COVID-19, the majority of which is for the development of new pharmaceutical products including vaccines, for clinical management and diagnostics. And most of these funded projects are taking place in developed countries, leaving Africa behind in terms of contributing to the scientific body of knowledge [16].

The focus of this study was on the research questions that will have an impact on the operational aspect of COVID-19 vaccine introduction. The target audience for this study are national immunisation program managers, national COVID-19 task forces, research and academic institutions, international agencies, donors, and national policy-makers who are in a position to launch or finance research projects to address locally relevant questions. We have excluded research questions related to vaccine effectiveness and immune response, as well as questions that can be easily addressed through routine data collection systems. In countries and contexts when the routine data collection systems and other programmatic review or documentation activities do not provide the required level of granularity of information, and when challenges of data completeness and data quality preclude adequate interpretation, it will be necessary to use cross-sectional operational research to bridge the gap and provide supplemental information required for decision-making. Our study has identified a menu of priority research questions across the different topic areas that focus on the most practical issues than can make a difference in terms of developing tailored solutions to improve COVID-19 vaccination coverage rates. The key priorities that came out in this study highlight that research should be focused on optimizing the delivery of existing interventions to maximize the impact on populations in need.

The respondents have mostly selected research questions in the area of demand generation and communication, as well as service delivery. This is understandable considering the significant challenges in generating sustained demand for COVID-19 vaccines as well as the need to develop flexible and tailored service delivery strategies that can reach priority target populations. Given the rapidly changing situation with the type and volumes of vaccine availability as well as the shifting public perception and demand, national immunisation programs and COVID-19 vaccination response taskforces should continue to identify and prioritize research questions with the greatest potential impact on country progress towards the vaccination targets. Countries should identify programmatic priorities, imbed research in their implementation planning, and mobilise funding as well as engage research and academic institutions as much as possible. In addition to establishing a research agenda, there is a need to ensure that research outputs are linked to policy making, resource allocation, operational planning and implementation.

This study has limitations. The number and scope of respondents was based on a convenience sample. Several identified resource persons could not take part in the study. The response rate was suboptimal, and more than half of the countries in the African Region were not represented among the respondents. With the relatively small sample, it was not possible to disaggregate the analysis to compare the prioritization provided by the respondents at different levels (country level versus subregional and regional levels). Our study was done with a view to get inputs for immediate guidance. However, we understand that the rating criteria and the framing of the research questions could be handled differently for a more detailed research prioritization process targeting a longer term period of implementation. This study was launched at the end of the first year of the introduction of COVID-19 vaccines. The initial 8 months of this first year were characterized by a shortage of vaccines in the majority of countries in the Region, and we expect the findings of this study to somehow reflect this reality. Considering the shifting demands and changes in the types and volumes of vaccine availability, we understand that the research priorities will continue to evolve.

## Conclusion

COVID-19 vaccination coverage rates remain very low within the African Region, as a result of multiple operational bottlenecks. High quality public health interventions using COVID-19 vaccines require investment in the delivery strategies and systems needed to generate demand and facilitate smooth service delivery. Countries should plan for and coordinate stakeholders to ensure that relevant operational research is done to respond to the top priority research questions, with a view to influence policies and implementation of strategies. Our study has helped to outline the critical operational research questions for countries and stakeholders at all levels to consider, as they prepare to scale up COVID-19 vaccination.

### What is known about this topic

- In the first year of the pandemic, a number of new COVID-19 vaccines have been introduced into national immunisation programs, targeting adult populations;
- As of December 2021, the level of coverage of COVID-19 vaccination remains very low in countries in the African Region of the WHO;
- National immunisation programs and COVID-19 response coordination bodies should continue to learn lessons, and update the local knowledge base in order to generate tailored solutions to challenges in vaccine introduction.

### What this study adds

- In the area of COVID-19 vaccination, numerous operational issues require in depth research to complement routine data collection systems, in order to generate practical solutions to evolving challenges;
- Research questions on the public knowledge and perceptions towards COVID-19 vaccines and vaccination, as well as on the most effective service delivery models are high priority at this point in the course of COVID-19 vaccine introduction. Other priority research questions have emerged from other program areas including planning and coordination, logistics, monitoring, vaccine safety and AEFI surveillance.
